# Allergy assessment in adult Portuguese cohort with eosinophilic esophagitis

**DOI:** 10.1186/2045-7022-5-S3-P106

**Published:** 2015-03-30

**Authors:** Rita Aguiar, João Marcelino, Fátima Duarte, Ana Célia Costa, Manuel A Pereira-Barbosa

**Affiliations:** 1 Immunoallergology Department, Hospital de Santa Maria, Lisbon, Portugal

## Introduction

Eosinophilic esophagitis (EoE) is an increasingly common cause of chronic and recurrent esophageal symptoms with significant impact on quality of life. Allergic responses to food and aeroallergens have been implicated in the etiology of this disease.

## Objectives

Clinical and sensitization profile characterization of an adult population with EoE diagnosis (EoEd) followed in our Immunoallergology Department.

## Methods

Retrospective study using our EoE database (2009-2014) of patients (pts) ≥ 18 years.

## Outcomes

Characterization of demographic, symptomatic, laboratorial [peripheral eosinophils (Eos), total and specific IgE], endoscopic and histological factors and sensitization profile (skin prick and patch tests to food and environmental allergens).

## Results

We included 25 patients (pts) [18M, 7F; 27±14, 7(18-70) years)]; median age of symptoms onset and diagnosis: 27(13-66) and 30(16-67) years respectively; average between symptoms onset and EoEd:27(1-180) months. Follow-up for 25.3±21.7(1-88) months. Reported symptoms-%pts: impaction-84,GERD-like symptoms 48, dysphagia-36, bloating-24, abdominal pain-16 and vomiting-12. Most frequent first symptom: impaction-48%pts. 60% of pts had personal history of atopy (respiratory disease-68% and food allergy-12%). Median peripheral Eos 388.8(40-980) and total IgE 163,8kU/L(5.6-436). The main baseline endoscopic findings (%pts): rings (43.5), furrows (26.1), hiatal hernia (21.7) and white plaques (21.7). 13% had microabcesses. After EoEd, allergic sensitization was identified in 88% pts (food allergens-72%, aeroallergens-60%, both-68%); food allergens [seafood (66.7), nuts (44.4), milk (22.2), egg (22.2), meat (22.2), cereals(16.7), fresh fruits(16.7) and soy (11.1)]; mites (54.2); pollens (37.5). After therapeutic: all had symptomatic improved; 12.5% pts achieved Eos≤15/HPF in esophageal biopsy, all with food allergy who underwent dietary eviction.

## Conclusions

The first and the most frequent symptom was food impaction. The prevalence of allergic sensitization was high. The potential severity of the symptoms justifies the importance of recognition and management of the disease. A multidisciplinary evaluation is essential in the clinical, diagnostic and therapeutic workup.

